# Circular RNAs in ferroptosis: regulation mechanism and potential clinical application in disease

**DOI:** 10.3389/fphar.2023.1173040

**Published:** 2023-06-02

**Authors:** Fei Li, Pei-Feng Li, Xiao-Dan Hao

**Affiliations:** Institute for Translational Medicine, The Affiliated Hospital of Qingdao University, College of Medicine, Qingdao University, Qingdao, China

**Keywords:** circular RNA, ferroptosis, regulation mechanism, clinical application, cancers

## Abstract

Ferroptosis, an iron-dependent non-apoptotic form of cell death, is reportedly involved in the pathogenesis of various diseases, particularly tumors, organ injury, and degenerative pathologies. Several signaling molecules and pathways have been found to be involved in the regulation of ferroptosis, including polyunsaturated fatty acid peroxidation, glutathione/glutathione peroxidase 4, the cysteine/glutamate antiporter system Xc-, ferroptosis suppressor protein 1/ubiquinone, and iron metabolism. An increasing amount of evidence suggests that circular RNAs (circRNAs), which have a stable circular structure, play important regulatory roles in the ferroptosis pathways that contribute to disease progression. Hence, ferroptosis-inhibiting and ferroptosis-stimulating circRNAs have potential as novel diagnostic markers or therapeutic targets for cancers, infarctions, organ injuries, and diabetes complications linked to ferroptosis. In this review, we summarize the roles that circRNAs play in the molecular mechanisms and regulatory networks of ferroptosis and their potential clinical applications in ferroptosis-related diseases. This review furthers our understanding of the roles of ferroptosis-related circRNAs and provides new perspectives on ferroptosis regulation and new directions for the diagnosis, treatment, and prognosis of ferroptosis-related diseases.

## 1 Ferroptosis

**FIGURE 1:**
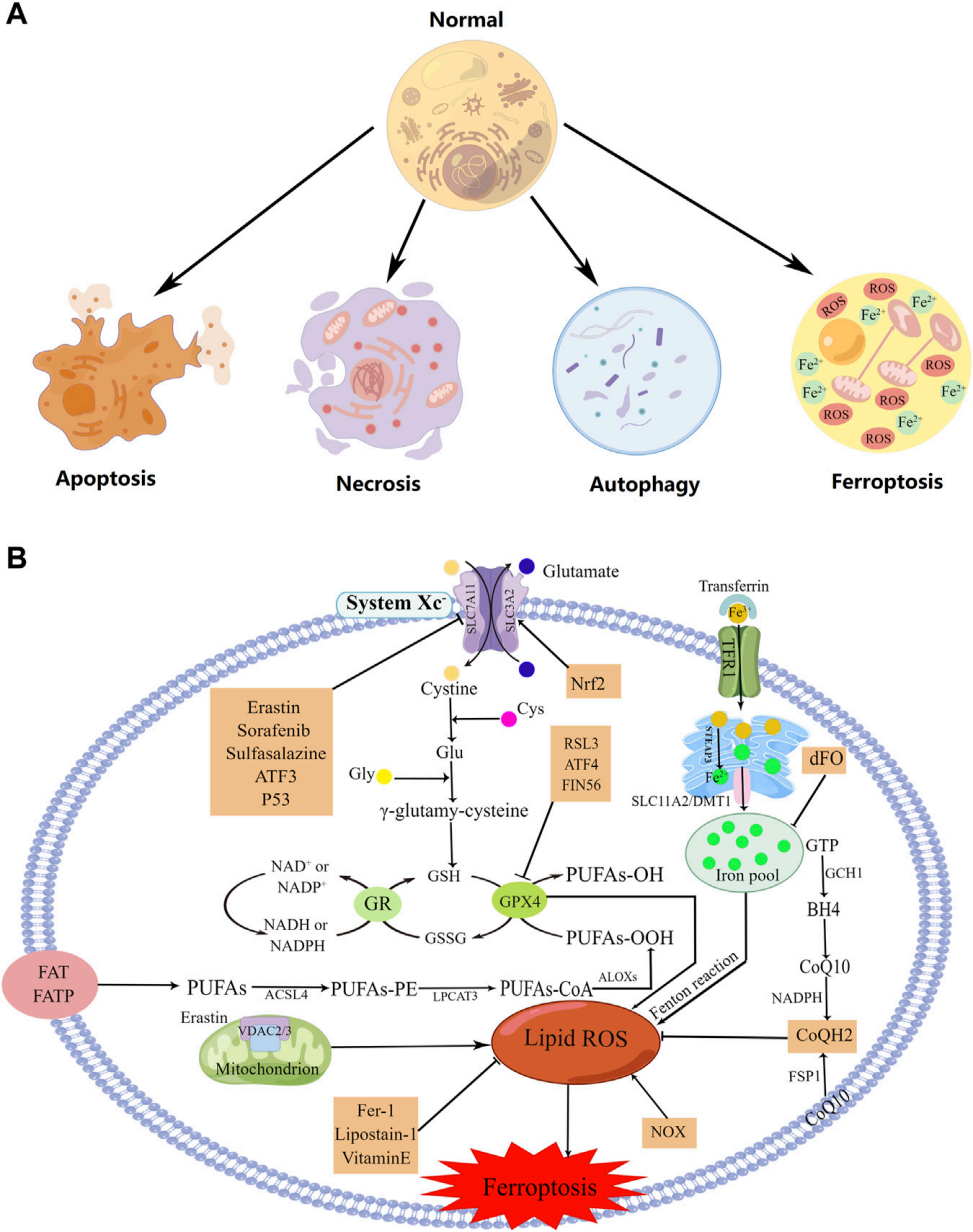
The regulation mechanism underlying cell ferroptosis. **(A)** Representations of ferroptosis, apoptosis, necrosis, and autophagy. **(B)** The regulation mechanism utilized during ferroptosis. Created using figdraw.

First described in 2012, ferroptosis is an iron- and reactive oxygen species (ROS)-dependent non-apoptotic form of regulatory cell death that differs from apoptosis, necrosis, and autophagy at the morphological, biochemical, and genetic levels (Figure 1A) (Dixon et al., 2012; Xie et al., 2016; Galluzzi et al., 2018). Morphologically, ferroptosis is characterized by marked mitochondrial contraction, increased membrane density, and the reduction or disappearance of mitochondrial cristae (Xie et al., 2016; Li et al., 2020). At the biochemical level, ferroptosis involves the accumulation of lipid peroxidation products and lethal ROS produced by iron metabolism, which can be inhibited by lipid peroxidation inhibitors and iron chelators, respectively (Xie et al., 2016; Galluzzi et al., 2018). Activation of mitochondrial voltage-dependent anion channels and mitogen-activated protein kinases, upregulation of endoplasmic reticulum (ER) stress, and inhibition of cystine/glutamate reverse transporters are all involved in the induction of ferroptosis (Xie et al., 2016).

Ferroptosis is involved in many physiological and pathological processes and is closely associated with many diseases, such as tumors, neurological disorders, ischemia-reperfusion injury, kidney injury, and blood diseases (Xie et al., 2016; Li et al., 2020). Several signaling molecules and pathways, such as polyunsaturated fatty acid (PUFA) peroxidation, glutathione (GSH)/glutathione peroxidase 4 (GPX4), the cysteine/glutamate antiporter (system Xc-), ferroptosis suppressor protein 1 (FSP1)/ubiquinone (CoQ10), and iron metabolism, have been found to be involved in ferroptosis regulation (Figure 1B) (Li et al., 2020). Also, four classes of ferroptosis inducers have been identified that drive cell death by either inhibiting system Xc-, inhibiting or degrading GPX4, consuming CoQ10, or inducing lipid peroxidation (Li et al., 2020).

### 1.1 Polyunsaturated fatty acid (PUFA) peroxidation

Lipid peroxidation is a hallmark of ferroptosis (Jiang et al., 2021). When subjected to oxidative or energy stress, cell membrane PUFAs—particularly arachidonic acid (AA) and adrenic acid—are oxidized to PUFA-OOH, inducing ferroptosis; the oxidation is catalyzed by acyl-CoA synthetase long-chain family member 4 (ACSL4), lysophosphatidylcholine acyltransferase (LPCAT), and lipoxygenases (ALOXs) (Figure 1B). PUFA peroxidation can cause the destruction of the lipid bilayer and damage cellular membranes, resulting in cellular dysfunction and cell death (Chen et al., 2021a).

Ferrostatin-1 (Fer-1) and lipostain-1 trap peroxides to reduce lipid peroxidation and alleviate ferroptosis (Ma et al., 2022a). In addition, fat-soluble vitamin E is adept at scavenging free radicals due to its high affinity for unpaired electrons (Ma et al., 2022a) and thus can inhibit ferroptosis mediated by lipid peroxidation (Hu et al., 2021).

### 1.2 Glutathione (GSH)/glutathione peroxidase 4 (GPX4)

Glutathione peroxidases (GPXs) protect cells against oxidative damage, thus preventing ferroptosis (Jiang et al., 2021). As a member of the GPX family, GPX4 can directly reduce peroxidized phospholipids in the cell membrane and is a pivotal regulator of ferroptosis (Yang et al., 2014; Jiang et al., 2021). More specifically, GPX4 inhibits ferroptosis by reducing each PUFA-OOH to the corresponding PUFA-OH and oxidizing GSH (a reductive cofactor of GPX4) to GSSG (oxidized GSH) (Figure 1B) (Ma et al., 2022a).

Overexpression or knockdown of GPX4 has been shown to affect the lethality of 12 ferroptosis inducers (Yang et al., 2014). Also, given that a decline in the level of GPX4 can lead to the accumulation of lipid peroxides and lead to ferroptosis, it is often used as a marker of ferroptosis (Yang et al., 2014; Jiang et al., 2021).

Ras-selective lethal small molecule 3 (RSL3) directly inhibits the activity of GPX4 by covalently binding to selenocysteine, which is located at the active site of GPX4, thereby inducing ferroptosis (Ma et al., 2022a). FIN56, another specific ferroptosis inducer, triggers ferroptosis by promoting the degradation of GPX4 via the acetyl-CoA pathway (Sun et al., 2021; Ma et al., 2022a). Activating transcription factor 4 (ATF4), a critical mediator of metabolic and oxidative homeostasis and cell survival (Chen et al., 2017a), inhibits GPX4 by activating heat shock 70 kDa protein 5 to bind to GPX4, thereby promoting ferroptosis (Zhu et al., 2017). FINO2 promotes ferroptosis via GPX4 inactivation and iron oxidation (Gaschler et al., 2018).

### 1.3 System Xc-

System Xc-is an important intracellular antioxidant system that is composed of two subunits: SLC7A11 and SLC3A2 (Chen et al., 2021a; Jiang et al., 2021; Du et al., 2022). SLC7A11 is responsible for the main transport activity and is highly specific for cystine and glutamate (Du et al., 2022). System Xc- exchanges intracellular glutamate for extracellular cystine (Cys2) at a 1:1 ratio, and the subsequent cystine-to-GSH reaction is catalyzed by glutamate cysteine ligase (GCL) and glutathione synthetase (GSS) (Chen et al., 2021a). Inhibiting the activity of system Xc- prevents the absorption of cystine, affects GSH synthesis, and subsequently reduces GPX4 activity (the membrane lipid-repair enzyme), thus reducing the cellular antioxidant capacity and promoting ferroptosis (Figure 1B) (Chen et al., 2021a; Du et al., 2022).

Activating transcription factor 3 (ATF3), a common stress sensor, promotes lipid peroxidation by inhibiting system Xc- (Wang et al., 2020a). Sorafenib (SF) is an oral tyrosine kinase inhibitor that induces GPX4 inactivation by blocking system Xc- and promotes ferroptosis (Zheng et al., 2021a). It has been shown that p53 decreases cystine uptake and intracellular GSH and induces ferroptosis by transcriptionally suppressing the expression of SLC7A11 (Ou et al., 2016). In addition, sulfadiazine has been shown to inhibit system Xc-, promote the accumulation of ROS, and induce ferroptosis (Yu et al., 2019), and NRF2 inhibits ferroptosis by increasing SLC7A11 (Song and Long, 2020).

### 1.4 Ferroptosis suppressor protein 1 (FSP1)/ubiquinone (CoQ10)

FSP1 is a GSH-independent ferroptosis suppressor encoded by apoptosis-inducing factor mitochondria-associated 2 (*AIFM2*) (Doll et al., 2019). It can suppress ferroptosis by acting on CoQ10: FSP1 reduces CoQ10 to ubiquinol (CoQH2) on the cell membrane, which acts as a free radical-trapping antioxidant to prevent lipid peroxidation on the cell membrane (Bersuker et al., 2019; Ma et al., 2022a). FSP1 can also catalyze CoQ10 regeneration by utilizing NAD(P)H (Doll et al., 2019). This GSH-independent FSP1/CoQ10/NAD(P)H pathway works in cooperation with the GPX4/GSH mechanism to suppress ferroptosis (Figure 1B) (Doll et al., 2019).

GTP loop hydrolase 1 (GCH1) is one of the rate-limiting enzymes involved in the synthesis of tetrahydrobiopterin (BH4) (Cronin et al., 2022), and GCH1 promotes the formation of CoQ10 and inhibits ferroptosis (Ma et al., 2022a).

### 1.5 Iron metabolism

Transferrin present in the serum binds to Fe^3+^, and the iron-loaded protein is recognized and bound by transferrin receptor protein 1 (TFR1) located on the cell membrane, forming a complex (Frazer and Anderson, 2014). Intracellular Fe^3+^ is reduced to Fe^2+^ by STEAP3 in the ER and then released by SLC11A2 into the cytoplasmic pool of free iron (Frazer and Anderson, 2014; Conrad et al., 2018). Fe^2+^ in the iron pool generates a considerable volume of hydroxyl radicals and ROS through the Fenton reaction, which causes ferroptosis (Figure 1B) (Frazer and Anderson, 2014; Conrad et al., 2018).

Deferoxamine (DFO) is an effective iron chelator (Zhu et al., 2022). After DFO enters the cell via endocytosis, it forms a stable octahedral coordination compound with Fe^3+^, thereby reducing the unstable iron pool in the cell (Ma et al., 2022a).

### 1.6 Mitochondria and transmembrane channels

Mitochondria play a key role in ferroptosis. ROS are derived in part from mitochondrial metabolism, and transmembrane voltage-dependent anion channels (VDACs) transport ions and metabolites across the outer mitochondrial membrane (Ma et al., 2022a). Erastin reduces mitochondrial membrane permeability through activation of VDAC2/3, thereby generating ROS that promote ferroptosis (Figure 1B) (DeHart et al., 2018; Ma et al., 2022a).

### 1.7 Chemical inducers/inhibitors of ferroptosis

Several chemicals have been shown to act as ferroptosis inducers or inhibitors (Du and Guo, 2022). As mentioned above, erastin induces ferroptosis by blocking VDACs, which affects GSH formation and oxidation (Du and Guo, 2022). Temozolomide induces ferroptosis by enhancing DMT1 (Du and Guo, 2022).Tertiary-butyl hydroperoxide and SF induce ferroptosis by affecting lipid metabolism and producing lipid ROS directly (Du and Guo, 2022). Brequinar inhibits tumor growth by inducing tumor cell ferroptosis (Du and Guo, 2022). Mison promotes ferroptosis by upregulating a GSH metabolic pathway regulator called dipeptidase-1, which increases cell sensitivity to ferroptosis (Du and Guo, 2022). Ciclopirox olamine, desferrioxamine, DFO, and deferasirox inhibit ferroptosis by sequestering iron ions (Du and Guo, 2022). Fer-1 and hydroquinone inhibit ferroptosis by inhibiting lipid oxidation (Du and Guo, 2022). In addition, 2-amino-5-chloro-N, 3-dimethylbenzamide can inhibit degradation of GPX4 and protect cells from the effects of ferroptosis (Du and Guo, 2022). Finally, alpha-tocopherol, the main component of vitamin E, can inhibit ferroptosis (Du and Guo, 2022).

## 2 The role of circular RNAs (circRNAs) in the regulation of ferroptosis

Circular RNA (circRNA) is a novel type of RNA that forms a covalently closed continuous loop with neither 5′-to-3′ polarity nor a polyadenylation tail (Chen and Yang, 2015; Qu et al., 2015). The unique circular structure of circRNA makes it more stable. It is formed by reverse splicing of pre-mRNA, and some circRNAs are abundant and evolutionarily conserved (Misir et al., 2022). *In vivo*, many circRNAs play important biological functions by acting as sponges for microRNAs, regulating protein functions, and self-translating (Gao et al., 2022; Misir et al., 2022). Increasing evidence suggests that circRNAs play important regulatory roles in the progression of many ferroptosis-related diseases and have great potential as novel diagnostic markers or therapeutic targets for such diseases (Zhang et al., 2020a; Liu et al., 2020; Xian et al., 2020; Xu et al., 2020; Li et al., 2021a; Wang et al., 2021a; Zhang et al., 2021a; Zhu et al., 2021a; Bazhabayi et al., 2021; Chen et al., 2021b; Wang et al., 2021b; Zhang et al., 2021b; Zheng et al., 2021b; Lyu et al., 2021; Shanshan et al., 2021; Wu et al., 2021; Yang et al., 2021; Yao et al., 2021; Chen et al., 2022a; Wang et al., 2022a; Wu et al., 2022a; Zhang et al., 2022a; Jiang et al., 2022; Jin et al., 2022; Mao and Liu, 2022; Ou et al., 2022; Pan et al., 2022; Yang et al., 2022). Therefore, in this review, we have summarized the recent research on ferroptosis-related circRNAs published prior to May 2022 in the PubMed and Web of Science databases (Table 1) to provide new perspectives on ferroptosis regulation and new directions for the diagnosis, treatment, and prognosis of ferroptosis-related diseases. The PubMed and Web of Science databases were searched using the keywords “ferroptosis” AND (“circRNA” OR “circular RNA” OR “non-coding RNA”). The resultant research studies were then manually collected and reviewed.

**TABLE 1:** Ferroptosis-related circular RNAs (circRNAs) associated with disease conditions.

Disease	CircRNA	Expression	References
Breast cancer (BC)	CircGFRA1	Upregulated in HER2-positive BC cells and tissues	Bazhabayi et al. (2021)
	Circ-BGN	Upregulated in trastuzumab-resistant BC cells and tissues	Wang et al. (2022a)
	CircRHOT1	BC cells	Zhang et al. (2021b)
Glioma	CircCDK14	Upregulated in glioma tissues and cells	Chen et al. (2022a)
	Circ-TTBK2		Zhang et al. (2020a)
Thyroid cancer	CircKIF4A	Upregulated in papillary thyroid cancer	Chen et al. (2021b)
	Circ_0067934	Upregulated in clinical thyroid cancer samples	Wang et al. (2021a)
Gastric cancer (GC)	Circ_0000190	Downregulated in GC tissues and cell lines	Jiang et al. (2022)
Lung cancer	CircP4HB	Upregulated in LUAD tissues	Pan et al. (2022)
	CircDTL	Upregulated in NSCLC tissues	Shanshan et al. (2021)
	CircRNA_101093	Upregulated in LUAD tissue and plasma exosome	Zhang et al. (2022a)
Hepatocellular carcinoma (HCC)	Hsa_circ_0008367	Most upregulated in sorafenib-treated HCC cells	Liu et al. (2020)
	Circ0097009	Upregulated in HCC tissues and cell lines	Lyu et al. (2021)
	CircIL4R	Upregulated in HCC tissues and cell lines	Xu et al. (2020)
Cervical cancer	CircLMO1	Downregulated in cervical cancer tissues and cell lines	Ou et al. (2022)
	CircEPSTI1	Upregulated in cervical cancer cell lines	Wu et al. (2021)
Colorectal cancer	Circ_0007142	Upregulated in colorectal cancer tissues and cell lines	Wang et al. (2021b)
	CircABCB10	Upregulated in rectal cancer tissues	Xian et al. (2020)
Oral squamous cell carcinoma (OSCC)	CircFNDC3B	Upregulated in clinical OSCC tissues	Yang et al. (2021)
Acute lymphoblastic leukemia (ALL)	Circ_0000745	Upregulated in the peripheral blood samples from ALL patients	Yang et al. (2022)
Esophageal cancer	CircPVT1	Upregulated in ESCC cells resistant to 5-FU	Yao et al. (2021)
Myocardial infarction (MI)	CircRNA1615	Downregulated in myocardial tissue of mice with MI	Li et al. (2021a)
Heart failure	CircSnx12	Downregulated in myocardial tissues of mice with TAC	Zheng et al. (2021b)
Acute cerebral infarction (ACI)	Circ-Carm1	Upregulated in the serum of patients with ACI	Mao and Liu (2022)
Traumatic brain injury (TBI)	CircPtpn14	Upregulated in the brain of patients and mice with TBI	Wu et al. (2022a)
Polycystic ovary syndrome (PCOS)	CircRHBG	Upregulated in granular cells of PCOS patients	Zhang et al. (2021a)
Diabetic nephropathy (DN)	Mmu_circRNA_0000309	Downregulated in podocytes of mice with DN	Jin et al. (2022)
Diabetic retinopathy (DR)	Circ-PSEN1	Upregulated in high glucose-treated ARPE19 cells	Zhu et al. (2021a)

**Notes:** NSCLC, non-small cell lung cancer; LUAD, lung adenocarcinoma; ESCC, esophageal squamous cell carcinoma; TAC, transverse aortic constriction; ARPE19, adult retinal pigment epithelial cell line-19.

### 2.1 Ferroptosis-inhibiting circRNAs

**FIGURE 2:**
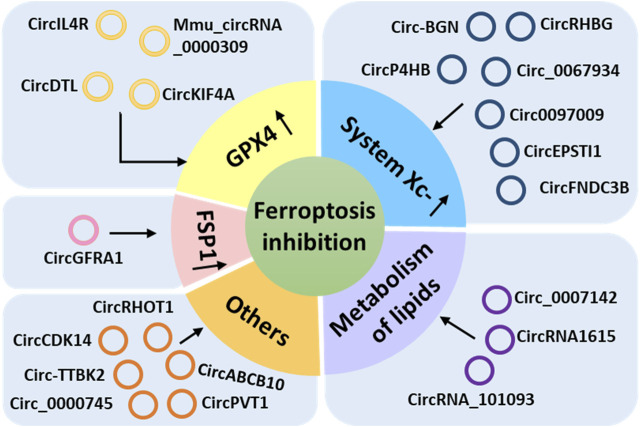
Ferroptosis-inhibiting circular RNAs classified according to the mechanism utilized to regulate ferroptosis.

More than 20 circRNAs have been reported to inhibit ferroptosis by acting on GPX4, system Xc-, FSP1, or lipid metabolism or other pathways and play important regulatory roles in the progression of many diseases (Zhang et al., 2020a; Xian et al., 2020; Xu et al., 2020; Li et al., 2021a; Wang et al., 2021a; Zhang et al., 2021a; Bazhabayi et al., 2021; Chen et al., 2021b; Wang et al., 2021b; Zhang et al., 2021b; Lyu et al., 2021; Shanshan et al., 2021; Wu et al., 2021; Yang et al., 2021; Yao et al., 2021; Chen et al., 2022a; Wang et al., 2022a; Zhang et al., 2022a; Jin et al., 2022; Pan et al., 2022; Yang et al., 2022), such as thyroid cancer, lung cancer, hepatocellular carcinoma (HCC), breast cancer, cervical cancer, oral squamous cell carcinoma (OSCC), glioma, colorectal cancer, esophageal cancer, diabetic nephropathy (DN), polycystic ovary syndrome (PCOS), acute lymphoblastic leukemia (ALL), and myocardial infarction (MI; Table 2). We classified these ferroptosis-inhibiting circRNAs according to the mechanism by which they regulate ferroptosis (Figure 2).

**TABLE 2:** The regulatory roles circular RNAs (circRNAs) play in disease progression via inhibiting ferroptosis.

Category	CircRNA	Mechanistic target	Function	Disease	References
GPX4 upregulation	CircKIF4A	miR-1231/GPX4	Inhibit ferroptosis and promote papillary thyroid cancer	Thyroid cancer	Chen et al. (2021b)
	CircDTL	miR-1287-5p/GPX4	Inhibit ferroptosis and promote non-small cell lung cancer	Non-small cell lung cancer	Shanshan et al. (2021)
	CircIL4R	miR-541-3p/GPX4	Inhibit ferroptosis and promote hepatocellular carcinoma	Hepatocellular carcinoma	Xu et al. (2020)
	Mmu_circRNA_0000309	miR-188-3p/GPX4	Inhibit ferroptosis and inhibit diabetic nephropathy	Diabetic nephropathy	Jin et al. (2022)
System Xc- upregulation	Circ-BGN	OTUB1/SLC7A11	Inhibit ferroptosis and promote HER-2-positive breast cancer	HER-2-positive breast cancer	Wang et al. (2022a)
	Circ_0067934	miR-545-3p/SLC7A11	Inhibit ferroptosis and promote thyroid cancer	Thyroid cancer	Wang et al. (2021a)
	CircP4HB	miR-1184/SLC7A11	Inhibit ferroptosis and promote lung adenocarcinoma	Lung adenocarcinoma	Pan et al. (2022)
	Circ0097009	miR-1261/SLC7A11	Inhibit ferroptosis and promote hepatocellular carcinoma	Hepatocellular carcinoma	Lyu et al. (2021)
	CircEPSTI1	miR-375/miR-409-3P/miR -515-5p/SLC7A11	Inhibit ferroptosis andpromote cervical cancer	Cervical cancer	Wu et al. (2021)
	CircFNDC3B	miR-520d-5p/SLC7A11	Inhibit ferroptosis and promote oral squamous cell carcinoma	Oral squamous cell carcinoma	Yang et al. (2021)
	CircRHBG	miR-515-5p/SLC7A11	Inhibit ferroptosis in polycystic ovary syndrome cells	Polycystic ovary syndrome	Zhang et al. (2021a)
FSP1 upregulation	CircGFRA1	miR-1228/AIFM2	Inhibit ferroptosis and promote HER-2-positive breast cancer	Breast cancer	Bazhabayi et al. (2021)
Lipid metabolism regulation	CircRNA_101093	Interacts with FABP3 and induce N-arachidonoyl taurine	Inhibit ferroptosis and Promote lung adenocarcinoma	Lung adenocarcinoma	Zhang et al. (2022a)
	Circ_0007142	miR-874-3p/GDPD5	Inhibit ferroptosis and promote colorectal cancer	Colorectal cancer	Wang et al. (2021b)
	CircRNA1615	miR-152-3p/LRP6	Inhibit ferroptosis and control pathological process of myocardial infarction	Myocardial infarction	Li et al. (2021a)
Inhibition of ferroptosis via other mechanisms	CircRHOT1	miR-106a-5p/STAT3	Inhibit ferroptosis and promote breast cancer	Breast cancer	Zhang et al. (2021b)
	CircCDK14	miR-3938/PDGFRA	Inhibit ferroptosis and promote glioma	Glioma	Chen et al. (2022a)
	Circ-TTBK2	miR-761/ITGB8	Inhibit ferroptosis and promote glioma	Glioma	Zhang et al. (2020a)
	CircABCB10	miR-326/CCL5	Inhibit ferroptosis and promote rectal cancer	Colorectal cancer	Xian et al. (2020)
	Circ_0000745	miR-494-3p/NET1	Inhibit ferroptosis and promote acute lymphoblastic leukemia	Acute lymphoblastic leukemia	Yang et al. (2022)
	CircPVT1	miR-30a-5p/FZD3	Inhibit ferroptosis and promote esophageal cancer	Esophageal cancer	Yao et al. (2021)

#### 2.1.1 CircRNAs that upregulate GPX4

Four circRNAs (circKIF4A, circDTL, circIL4R, and mmu_circRNA_0000309) inhibit ferroptosis by upregulating GPX4 (Figure 2; Table 2). CircKIF4A reportedly promotes the malignant progression of papillary thyroid cancer and inhibits ferroptosis by sponging miR-1231 and then upregulating its target gene *GPX4* (Chen et al., 2021b). Silencing of circKIF4A can downregulate GPX4, resulting in the proliferation and metastatic inhibition of papillary thyroid cancer cells and inhibition of tumor growth *in vivo* (Chen et al., 2021b). CircDTL inhibits ferroptosis and apoptosis of non-small cell lung cancer (NSCLC) cells through the circDTL/miR1287-5p/GPX4 axis (Shanshan et al., 2021). Downregulation of circDTL was found to increase cellular ROS, malondialdehyde (MDA; an endogenous genotoxic product of lipid peroxidation), and Fe^2+^ levels and reduce GSH levels, thus promoting ferroptosis of NSCLC cells (Shanshan et al., 2021). CircIL4R positively regulates the expression of GPX4 by adsorbing miR-541-3p, facilitates tumorigenesis, and inhibits ferroptosis in HCC cells (Xu et al., 2020). Knockdown of circIL4R can aggravate erastin-induced ferroptosis by increasing iron accumulation and oxidative stress in HCC cells, hindering the carcinogenesis process. Mmu_circRNA_0000309 was found to inhibit ferroptosis-dependent mitochondrial damage and podocyte apoptosis by competitively adsorbing miR-188-3p to promote GPX4 expression, thereby participating in the improvement of DN mediated by germacrone (Jin et al., 2022). Germacrone is the main bioactive component of turmeric, which has anti-inflammatory and antioxidant effects (Aggarwal et al., 2013). Silencing mmu_circRNA_0000309 or introducing miR-188-3p mimics was found to eliminate the anti-apoptotic and anti-injury effects of germacrone by aggravating mitochondrial damage and increasing the levels of ROS and iron deposition-related proteins (Jin et al., 2022). In the same study, overexpression of GPX4 was found to neutralize mitochondrial damage and ferroptosis mediated by mmu_circRNA_0000309 silencing (Jin et al., 2022).

#### 2.1.2 CircRNAs that upregulate system Xc-

SLC7A11, a core subunit of system Xc-, imports cystine into the cell for GSH biosynthesis and as an antioxidant defense (Koppula et al., 2021). Seven circRNAs (circ-BGN, circ_0067934, circP4HB, circ0097009, circEPSTI1, circFNDC3B, and circRHBG) have been reported to inhibit ferroptosis via upregulation of SLC7A11 (Figure 2; Table 2).

OTU deubiquitinase, ubiquitin aldehyde binding 1 (OTUB1) is a highly expressed cysteine protease and a member of the deubiquitinating enzyme family (Liu et al., 2014; Que et al., 2020). Circ-BGN was found to directly bind to OTUB1 and SLC7A11 and enhance OTUB1-mediated deubiquitination of SLC7A11, thereby inhibiting ferroptosis (Wang et al., 2022a). Downregulation of circ-BGN significantly increases the levels of lipid ROS, MDA, and Fe^2+^, inhibits GPX4 activity, and leads to the inhibition of activity in breast cancer cells (Wang et al., 2022a). In addition, circ-BGN knockdown has been shown to enhance the significant inhibition of cell growth mediated by erastin on trastuzumab resistance breast cancer cells (Wang et al., 2022a).

Circ_0067934 reportedly upregulates the expression of SLC7A11 and thus promotes the progression of thyroid cancer and inhibits ferroptosis in thyroid cancer cells by adsorbing miR-545-3p (Wang et al., 2021a). Silencing circ_0067934 decreased the cell survival rate and enhanced ferroptosis and apoptosis in thyroid cancer cells (Wang et al., 2021a). Overexpression of an miR-545-3p inhibitor or SLC7A11 rescued the inhibitory effect of silencing circ_0067934 on thyroid cancer cells and resulted in a decrease in the levels of ferroptosis-associated markers, such as Fe^2+^, iron, and ROS (Wang et al., 2021a).

CircP4HB, which is also called hsa_circ_0046263, is derived from the alternative transcription of the prolyl 4-hydroxylase subunit beta gene (Wang et al., 2019a). In lung adenocarcinoma (LUAD) cells, circP4HB was found to direct ferroptosis by regulating miR-1184/SLC7A11-mediated GSH synthesis (Pan et al., 2022). CircP4HB targeted and sponged miR-1184, and *SLC7A11* was found to be a target gene of miR-1184 (Pan et al., 2022). As an inhibitor of ferroptosis, circP4HB protects LUAD cells from ferroptosis by triggering GSH synthesis (Pan et al., 2022).

In HCC cells, SLC7A11 was found to be regulated by circ0097009 via the sponging of miR-1261. Ferroptosis is involved in HCC progression through the circ0097009/miR-1261/SLC7A11 axis (Lyu et al., 2021). Downregulation of circ0097009 has been shown to significantly inhibit cell growth, invasion, and metastasis and promote ferroptosis in HCC cells (Lyu et al., 2021).

CircEPSTI1, also known as hsa_circRNA_000479, is a cancer-associated circRNA (Peng et al., 2020; Tan et al., 2020; Xie et al., 2020). As a competing endogenous RNA (ceRNA), circEPSTI1 upregulates the expression of SLC7A11 by adsorbing miR-375, miR-409-3p, and miR-515-5p in cervical cancer cells (Wu et al., 2021). Silencing of circEPSTI1 inhibited cervical cancer cell proliferation and induced SLC7A11-mediated ferroptosis, and overexpression of SLC7A11 reversed this effect (Wu et al., 2021).

CircFNDC3B, also known as circ_0006156, has biological functions in a variety of cancers, such as papillary thyroid cancer (Wu et al., 2020), esophageal squamous cell carcinoma (ESCC) (Tang et al., 2022), and gastric cancer (GC) (Hong et al., 2019). A recent study found that circFNDC3B protects OSCC cells from ferroptosis and promotes malignant progression by regulating the miR-520d-5p/SLC7A11 axis (Yang et al., 2021). CircFNDC3B can enhance the accumulation of ROS, iron, and Fe^2+^ in cells to inhibit ferroptosis (Yang et al., 2021). Knockdown of circFNDC3B has been shown to enhance the inhibitory effect of erastin on OSCC cells, thereby inducing ferroptosis in OSCC cells (Yang et al., 2021).

CircRHBG is involved in the proliferation and ferroptosis of PCOS granulosa cells through the miR-515/SLC7A11 axis (Zhang et al., 2021a). In PCOS cells, circRHBG acts as a ceRNA for miR-515 and upregulates SLC7A11 (Zhang et al., 2021a). The downregulation of circRHBG was found to promote ferroptosis by causing a decrease in the GSH-to-GSSG ratio, leading to GPX4 inactivation (Zhang et al., 2021a).

#### 2.1.3 CircRNAs that upregulate FSP1

CircGFRA1 acts as a ceRNA for miR-1228 and upregulates *AIFM2*, which encodes FSP1 (a ferroptosis suppressor that acts via CoQ10) (Bazhabayi et al., 2021). CircGFRA1 has been shown to promote the progression of HER2-positive breast cancer via the miR-1228/*AIFM2* axis (Bazhabayi et al., 2021). The silencing of circGFRA1 can enhance ferroptosis through the circGFRA1/miR-1228/*AIFM2* axis (Bazhabayi et al., 2021) and inhibit the proliferation, infiltration, and metastasis of HER2-positive breast cancer cells (Bazhabayi et al., 2021). In addition, circGFRA1 silencing also leads to a decrease in the GSH-to-GSSG ratio and downregulation of GPX4; the decrease in the GSH-to-GSSG ratio results in GPX4 inactivation, further promoting lipid ROS accumulation and ferroptosis (Bazhabayi et al., 2021).

#### 2.1.4 CircRNAs that regulate lipid metabolism

Some circRNAs that are involved in lipid metabolism have been reported to inhibit ferroptosis (Li et al., 2021a; Wang et al., 2021b; Zhang et al., 2022a) (Figure 2). It was found that circRNA_101093 can desensitize LUAD cells to ferroptosis by upregulating fatty acid-binding protein 3 (*FABP3*), reducing global AA, and preventing AA incorporation into the plasma membrane (Zhang et al., 2022a). CircRNA_101093 integrated with and increased FABP3, which then transported AA and facilitated its reaction with taurine, thus reducing global AA and inducing production of N-arachidonoyl taurine (NAT; the product of AA and taurine) (Zhang et al., 2022a). NAT plays a role in desensitizing cells to ferroptosis by downregulating the expression of related enzymes (i.e., ACSL4, LPCAT3, and PLTP) and preventing the incorporation of AA into the plasma membrane of LUAD cells (Du et al., 2019; Cui et al., 2021; Jiang and Yu, 2021).

Altered choline phospholipid metabolism is a hallmark of cancer (Cao et al., 2012). Glycerophosphodiester phosphodiesterase domain containing 5 (*GDPD5*), the target gene of miR-874-3p, encodes a glycerophosphodiester phosphodiesterase that catalyzes the hydrolysis of deacylated glycerophospholipids to glycerol phosphate and an alcohol (Lang et al., 2008). Circ_0007142 has been identified as a carcinogenic factor due to its ability to regulate tumorigenesis and ferroptosis in colorectal cancer cells via the miR-874-3p/GDPD5 axis (Wang et al., 2021b). Low expression of circ_0007142 can inhibit proliferation and promote apoptosis and ferroptosis in colorectal cancer cells (Wang et al., 2021b).

Lipoprotein receptor-related protein-6 (LRP6) is involved in lipid homeostasis and is an essential co-receptor for canonical Wnt signaling (Li et al., 2010). It has been found that circRNA1615 regulates the expression of *LRP6* through the adsorption of miR-152-3p to prevent LRP6-mediated autophagy-related ferroptosis in cardiomyocytes, ultimately controlling the pathological process of MI (Li et al., 2021a). In addition, higher levels of MDA and Fe^2+^ observed in MI tissues have suggested that ferroptosis occurs in cardiomyocytes (Li et al., 2021a). LRP6 interference increased the expression of the autophagy-related proteins LC3-A/B (microtubule-associated protein 1 light chain 3-A/B) and autophagy related 5 and decreased the expression of sequestosome 1, resulting in induced ferroptosis in cardiomyocytes through autophagy (Li et al., 2021a).

#### 2.1.5 CircRNAs that inhibit ferroptosis via other pathways

Some circRNAs have also been reported to inhibit ferroptosis via signal transducer and activator of transcription 3 (STAT3), platelet derived growth factor receptor alpha (PDGFRA), integrin subunit beta 8 (ITGB8), and other pathways and play important regulatory roles in the progression of various cancers, such as breast cancer, glioma, lung cancer, HCC, colorectal cancer, ALL, and esophageal cancer (Zhang et al., 2020a; Xian et al., 2020; Zhang et al., 2021b; Yao et al., 2021; Chen et al., 2022a; Yang et al., 2022) (Table 2; Figure 2).

CircRHOT1 has been found to play a key role in the development of multiple types of diseases, such as HCC (Wang et al., 2019b), osteoarthritis (Man et al., 2022), and NSCLC (Ren et al., 2021). In breast cancer cells, circRHOT1 functions by adsorbing miR-106a-5p, which targets STAT3 in this cell type (Zhang et al., 2021b). CircRHOT1 was found to promote the proliferation and migration of breast cancer cells and inhibit apoptosis and ferroptosis through the miR-106a-5p/STAT3 axis (Zhang et al., 2021b).

The transmembrane receptor PDGFRA is overexpressed, amplified, mutated, or truncated in gliomas and is the second most frequently mutated tyrosine kinase receptor in glioblastomas (Alentorn et al., 2012; Higa et al., 2022). It has been found that circCDK14 sponges miR-3938 and upregulates PDGFRA expression, resulting in resistance to ferroptosis and promotion of glioma progression (Chen et al., 2022a). In the same study, when circCDK14 was deleted, the SLC7A11 and GPX4 levels were significantly reduced and the Fe^2+^ and ROS levels were significantly increased (Chen et al., 2022a). In addition, circCDK14 has also been shown to promote epithelial-mesenchymal transition in glioma cells by regulating PDGFRA expression (Chen et al., 2022a).

Another study revealed that circ-TTBK2, also named has_circ_0000594, regulates glioma cell proliferation, invasion, and ferroptosis through the miR-761/ITGB8 axis (Liao et al., 2015; Zhang et al., 2020a). Knockdown of circ-TTBK2 or increased expression of miR-761 was found to delay the proliferation and invasion of glioma cells and promote ferroptosis (Zhang et al., 2020a). *ITGB8* encodes a beta subunit of integrin (integrin beta 8) (He et al., 2018) and is the target gene of miR-761; its overexpression can restore the inhibitory effect of miR-761 on cell proliferation (Zhang et al., 2020a).

CircABCB10, also known as circRNA-0008717 (Tian et al., 2019), plays a key role in the progression of many tumors, such as GC (Zhang et al., 2021c), HCC (Fu et al., 2019), and NSCLC (Tian et al., 2019). Xian et al. (Xian et al., 2020) found that circABCB10 acts as a sponge for miR-326, regulating C-C motif chemokine ligand 5 (CCL5) expression in rectal cancer cells (Xian et al., 2020). The deletion of circABCB10 significantly promoted the accumulation of intracellular lipid ROS and Fe^2+^. CircABCB10 regulates ferroptosis and apoptosis in rectal cancer cells through the miR-326/CCL5 axis (Xian et al., 2020).

Oncogenic neuroepithelial cell transforming 1(*NET1*), which lacks the first 145 amino acids, is present in the cytosol and contributes to the efficient activation of RhoA and the formation of actin stress fibers in many tumor cell types (Wei et al., 2017). Circ_0000745 was found to inhibit ferroptosis and promote the progression of acute lymphoblastic leukemia via the miR-494-3p/NET1 axis (Yang et al., 2022). Circ_0000745 interference has also been shown to inhibit the cell cycle and glycolysis and increase the levels of intracellular iron and lipid ROS induced by erastin, thus accelerating ferroptosis (Yang et al., 2022). Silencing miR-4943p, the target of circ_0000745, largely reduced the antitumor effect induced by silencing circ_0000745 (Yang et al., 2022). It was also found that overexpression of NET1, the target of miR-494-3p, could partially reverse the antitumor effect induced by miR-494-3p overexpression (Yang et al., 2022).

5-fluorouracil (5-FU) is a typical antitumor drug, and circPVT1 has been found to inhibit the chemoresistance of ESCC cells to 5-FU by influencing ferroptosis and the Wnt/b-catenin pathway via the miR-30a-5p/Frizzled3 (FZD3) axis (Yao et al., 2021). Knockdown of circPVT1 can inhibit the Wnt/b-catenin pathway in ESCC cells, significantly increase the expression levels of ROS and ferroptosis-associated parameters, and significantly reduce the expression of GSH, GPX4, and SLC7A11; these effects can be significantly reversed by the addition of an miR-30a-5p inhibitor and by FZD3 overexpression (Yao et al., 2021).

### 2.2 Ferroptosis-stimulating circRNAs

**FIGURE 3:**
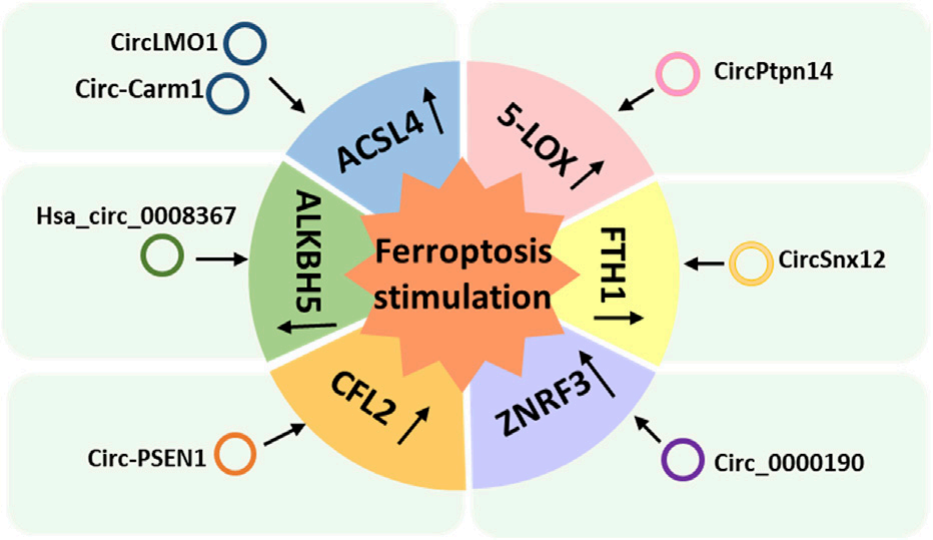
Ferroptosis-stimulating circular RNAs classified according to the mechanism used to regulate ferroptosis.

Seven circRNAs have been identified that can stimulate ferroptosis via various pathways and that play important regulatory roles in the progression of many diseases, including cervical cancer, acute cerebral infarction (ACI), traumatic brain injury (TBI), heart failure (HF), diabetic retinopathy, HCC, and GC (Liu et al., 2020; Zhu et al., 2021a; Zheng et al., 2021b; Wu et al., 2022a; Jiang et al., 2022; Mao and Liu, 2022; Ou et al., 2022) (Table 3). We classified these ferroptosis-stimulating circRNAs according to the mechanism by which they regulate ferroptosis (Figure 3).

**TABLE 3:** The regulatory roles circular RNAs (circRNAs) play in disease progression via stimulating ferroptosis.

Category	CircuRNA	Mechanistic target	Function	Disease	References
ACSL4 upregulation	CircLMO1	miR-4291/ACSL4	Promote ferroptosis and inhibit cervical cancer	Cervical cancer	Ou et al. (2022)
	Circ-Carm1	miR-3098-3p/ACSL4	Promote ferroptosis in acute cerebral infarction	Acute cerebral infarction	Mao and Liu (2022)
5-lipoxygenase upregulation	CircPtpn14	miR-351-5p/5-LOX	Promote ferroptosis and reverse the effects of melatonin	Traumatic brain injury	Wu et al. (2022a)
Ferritin heavy chain 1 upregulation	CircSnx12	miR-224-5p/FTH1	Promote ferroptosis and lead to cardiomyocyte death	Heart failure	Zheng et al. (2021b)
Cofilin-2 upregulation	Circ-PSEN1	miR-200b-3p/CFL2	Promote ferroptosis and involved in Diabetic retinopathy	Diabetic retinopathy	Zhu et al. (2021a)
Inhibition of ALKBH5-mediated autophagy inhibition	Hsa_circ_0008367	Interacts with ALKBH5	Promote ferroptosis and inhibit hepatocellular carcinoma	Hepatocellular carcinoma	Liu et al. (2020)
ZNRF3 upregulation	Circ_0000190	miR-382-5p/ZNRF3	Promote ferroptosis and inhibit gastric cancer	Gastric cancer	Jiang et al. (2022)

#### 2.2.1 CircRNAs that upregulate acyl-CoA synthetase long-chain family member 4

ACSL4 is an isozyme of the long-chain fatty-acid-coenzyme A ligase family and preferentially activates PUFAs for phospholipid biosynthesis and for fueling ferroptosis; hence, it is a typical marker of ferroptosis (Zhang et al., 2022b). CircLMO1 and circ_Carm1 have been reported to stimulate ferroptosis by upregulating ACSL4 (Figure 3) (Mao and Liu, 2022; Ou et al., 2022). CircLMO1, also known as hsa_circ_ 0021087, acts as a ceRNA and upregulates ACSL4 expression by adsorbing miR-4192, which decreases GSH and MDA and increases ROS levels, stimulating ferroptosis (Ou et al., 2022). Overexpression of circLMO1 inhibits cervical cancer growth and metastasis both *in vitro* and *in vivo*, whereas circLMO1 depletion promotes cervical cancer cell proliferation and invasion (Ou et al., 2022).

Circ-Carm1 is involved in the progression of ACI; it induces miR-3098-3p to upregulate the expression of ACSL4 *in vitro* (Mao and Liu, 2022). Knockdown of circ-Carm1 was shown to restore cell viability and inhibit ferroptosis; however, downregulation of miR-3098-3p could reverse the inhibitory effect of circ-Carm1 by promoting the secretion of Fe^2+^ and MDA (Mao and Liu, 2022). ACSL4 is the target of miR-3098-3p (Mao and Liu, 2022). Upregulation of ACSL4 inhibited the effect of miR-3098-3p on cell viability and ferroptosis (Mao and Liu, 2022).

#### 2.2.2 A circRNA that upregulates 5-lipoxygenase

5-lipoxygenase (5-LOX), a member of the lipoxygenase gene family, plays an important role in producing toxic lipids; thus, induces ferroptosis. A study showed that melatonin reduced ferroptosis and ER stress in TBI by regulating the expression of ferroptosis-related 5-LOX through the circPtpn14/miR-351-5p/5-LOX signaling pathway (Wu et al., 2022a). Overexpression of circPtpn14 can partially abolish the inhibitory effect of melatonin on ferroptosis and reverse the anti-lipid peroxidation and anti-ER stress effects of melatonin (Wu et al., 2022a). The introduction of miR-351-5p (the target of circPtpn14) was found to reverse the 5-LOX upregulation and ER stress signaling activation caused by circPtpn14 overexpression alone and to rescue the decreased cell viability, inhibition of GPX activity, and increased blood-brain barrier permeability *in vitro* caused by circPtpn14 Wu et al. 2022a.

#### 2.2.3 A circRNA that upregulates ferritin heavy chain 1

Zheng et al. (2021b) proposed that circSnx12 is involved in ferroptosis during HF by targeting the miR-224-5p/ferritin heavy chain 1 (FTH1) axis. FTH1 is a ferritin complex that catalyzes the conversion of Fe^2+^ into Fe^3+^ to protect the cell from oxidative damage (Zhang et al., 2017). CircSnx12 acts as a sponge for miR-224-5p, and *FTH1* is a target gene of miR-224-5p. Low expression of circSnx12 and high expression of miR-224-5p can downregulate FTH1 expression, which can directly induce ferroptosis in cardiomyocytes and eventually lead to cardiomyocyte death (Zheng et al., 2021b).

#### 2.2.4 A circRNA that upregulates cofilin-2

Circ-PSEN1, also known as circ_0008521, regulates ferroptosis in retinal pigment epithelial cells of patients with diabetic retinopathy (DR) via the miR-200b-3p/cofilin-2 (CFL2) axis (Zhu et al., 2021a). CFL2 is a small actin-binding protein and a member of the AC group of proteins, which is predominantly expressed at sarcomeres in skeletal and cardiac muscles (Agrawal et al., 2012). Circ-PSEN1 acts as a sponge for miR-200b-3p, and *CFL2* is a target gene of miR-200b-3p (Zhu et al., 2021a). Knockdown of circ-PSEN1 was found to increase cell viability and inhibit ferroptosis, and CFL2 was found to abolish the inhibitory effect of miR-200b-3p on ferroptosis (Zhu et al., 2021a). Overexpression of *CFL2* resulted in a decrease in GSH and an increase in MDA and ferrous iron, which decreased cell viability (Zhu et al., 2021a).

#### 2.2.5 A circRNA that induces ferroptosis by interacting with AlkB homologue 5

Hsa_circ_0008367, also known as cIARS, is a promoter of ferroptosis in HCC cells treated with SF (Liu et al., 2020). SF has been approved by the US Food and Drug Administration for the treatment of HCC. However, its clinical application is limited by its poor water solubility and adverse side effects (Thapa et al., 2015). Nevertheless, cIARS expression was found to be significantly upregulated in SF-treated HCC cells, and cIARS positively regulates SF-induced ferroptosis by inhibiting AlkB homologue (ALKBH) 5-mediated autophagy inhibition (Liu et al., 2020). AlkB homologues are a specific family of demethylases that depend on Fe^2+^ and α-ketoglutarate to catalyze demethylation of different substrates (Xu et al., 2021). ALKBH5 is a negative regulator of autophagy in HCC cells, and cIARS can inhibit the activity of ALKBH5 in the regulation of autophagy (Liu et al., 2020).

#### 2.2.6 A circRNA that induces ferroptosis by upregulating zinc and ring finger 3 (ZNRF3)

The tumor suppressor circ_0000190 sponges miR-382-5p and suppresses cell proliferation and motility and promotes cell death by targeting ZNRF3 in GC cells (Jiang et al., 2022). ZNRF3 is a transmembrane E3 ubiquitin ligase that inhibits endogenous Wnt-mediated activation of the β-catenin signaling pathway (Hao et al., 2012). Circ_0000190 induces apoptosis and ferroptosis in GC cells (Jiang et al., 2022). Overexpression of circ_0000190 was found to significantly increase the levels of iron and Fe^2+^ in GC cells treated with erastin or RSL3 (Jiang et al., 2022). Additionally, with the accumulation of circ_0000190, the production of MDA and lipid ROS was found to increase, and the activity of caspase-3 and the rate of apoptosis also increased significantly (Jiang et al., 2022). As a target of circ_0000190, miR-382-5p has a negative regulatory relationship with circ_0000190 (Jiang et al., 2022). Meanwhile, ZNRF3 is the target of miR-382-5p, and overexpression of it can also counteract the effect of miR-382-5p accumulation on GC cells (Jiang et al., 2022).

### 2.3 CircRNAs that are potential biomarkers of ferroptosis

Several studies have reported that a range of circRNAs exhibit abnormal expression levels in cells treated with ferroptosis inducers (Liu et al., 2020; Wang et al., 2022a; Hou et al., 2022; Mao and Liu, 2022). For example, compared with untreated HCC cell lines, 102 significantly upregulated circRNAs were identified in cells treated with the ferroptosis inducer SF (Liu et al., 2020). The circRNA that recorded the highest level of upregulation in that study, hsa_circ_0008367, has great potential as a biomarker of ferroptosis induced by SF. In another study, circ-Carm1 was highly expressed in HT22 cells after treatment with erastin, a ferroptosis activator (Mao and Liu, 2022). Yet another study found that erastin-treated HER2-positive breast cancer cells presented significantly high expression levels of circ-COL1A2, circ-SC5D, circ-MSH2, circ-ACRBP, and circ-DTL compared with untreated cells (Wang et al., 2022a). Furthermore, RNA sequencing was used to identify 17 downregulated and 18 upregulated circRNAs in human coronary artery endothelial cells after hydrogen peroxide treatment, and the five most upregulated circRNAs were hsa_circ_0001558, hsa_circ_0002665, hsa_circ_0000530, hsa_circ_0005871, and hsa_circ_0009353 (Hou et al., 2022).

CircRNAs that are highly expressed in cells after treatment with a ferroptosis inducer have potential as biomarkers of ferroptosis. Their identification also provides new avenues for the detection of ferroptosis *in vivo* or *in vitro*. However, further studies are needed to confirm the potential applications of ferroptosis-related circRNAs as biomarkers *in vivo* and *in vitro*.

## 3 Potential clinical applications of circRNAs in the diagnosis and treatment of ferroptosis-related diseases

### 3.1 Breast cancer

In 2020, breast cancer was the most commonly diagnosed cancer in women, and it is the fifth leading cause of cancer deaths worldwide (Sung et al., 2021). Early diagnosis and timely treatment are vital for improving the prognosis of breast cancer patients. Several studies have suggested that ferroptosis-related circRNAs can be used as biomarkers for the diagnosis, treatment, and prognosis of breast cancer (Bazhabayi et al., 2021; Zhang et al., 2021b; Wang et al., 2022a) (Table 4).

**TABLE 4:** Potential therapeutic target and diagnostic and prognostic biomarkers of diseases.

Disease	Diagnostic biomarker	Therapeutic target	Prognostic biomarker
Breast cancer	CircGFRA1	CircGFRA1;	Circ-BGN
		Circ-BGN;	
		CircRHOT1	
Glioma	CircCDK14;	CircCDK14;	CircCDK14
	Circ-TTBK2	Circ-TTBK2	
Thyroid cancer	CircKIF4A;	CircKIF4A;	
	Circ_0067934	Circ_0067934;	
Gastric cancer	Circ_0000190	Circ_0000190	Circ_0000190
Lung cancer	CircDTL;	CircDTL;	CircP4HB;
	CircP4HB;	CircP4HB;	
	CircRNA_101093	CircRNA_101093	
Hepatocellular carcinoma	CircIL4R;	CircIL4R;	CircIL4R
	Circ0097009	Circ0097009;	
		Hsa_circ_0008367	
Cervical cancer	CircEPSTI1;	CircEPSTI1;	CircLMO1
	CircLMO1;	CircLMO1;	
Colorectal cancer	Circ_0007142;	Circ_0007142;	
	CircABCB10	CircABCB10	
Oral squamous cell carcinoma	CircFNDC3B	CircFNDC3B	CircFNDC3B
Esophageal cancer	CircPVT1	CircPVT1	
Acute lymphoblastic leukemia	Circ_0000745	Circ_0000745	
Myocardial infarction		CircRNA1615	
Heart failure	CircSnx12	CircSnx12	
Acute cerebral infarction	Circ-Carm1	Circ-Carm1	
Traumatic brain injury		CircPtpn14	
Polycystic ovary syndrome	CircRHBG	CircRHBG	
Diabetic nephropathy		Mmu_circRNA_0000309	
Diabetic retinopathy		Circ-PSEN	

CircGFRA1 has great potential as a diagnostic marker and therapeutic target for HER2-positive breast cancer. The expression of circGFRA1 is significantly upregulated in HER2-positive breast cancer tissues compared with non-malignant tissues (Bazhabayi et al., 2021). Furthermore, deletion of circGFRA1 could delay tumor growth *in vivo* (Bazhabayi et al., 2021). Circ-BGN has potential as a therapeutic target and a prognostic biomarker for trastuzumab-resistant breast cancer (Wang et al., 2022a). The expression of circ-BGN is significantly increased in trastuzumab-resistant breast cancer cells and tissues compared to parental cells, and its increase is associated with poor overall survival (Wang et al., 2022a). In addition, circRHOT1 promotes tumor growth by inhibiting ferroptosis in breast cancer cells and is thus a promising therapeutic target for the development of future breast cancer treatment strategies (Zhang et al., 2021b).

### 3.2 Glioma

Glioma is the most common type of primary intracranial tumor in adults; it can occur anywhere in the central nervous system and is associated with high mortality and morbidity rates (Morgan, 2015). The identification of ferroptosis-related circRNAs is providing new directions for research on the diagnosis and treatment of gliomas (Table 4).

CircCDK14 resists ferroptosis and promotes tumor progression; thus, it may form part of a therapeutic strategy and holds promise as a diagnostic and prognostic biomarker for glioma (Chen et al., 2022a). Glioma tissues have significantly higher levels of circCDK14 expression than normal tissues, and the expression level is inversely related to the overall survival time of glioma patients: the higher the circCDK14 expression, the worse the prognosis of the glioma patient. Grade III–IV glioma tissues have significantly higher levels of circCDK14 than grade I–II glioma tissues (Chen et al., 2022a). CircCDK14 silencing has been found to reduce the growth of tumors *in vivo* (Chen et al., 2022a). Furthermore, circ-TTBK2 is upregulated in glioma tissues (Zhang et al., 2020a), and it regulates glioma cell proliferation, invasion, and ferroptosis, which means that it could form the basis of a therapeutic strategy and potentially be used as a diagnostic biomarker for glioma as well. The deletion of circGFRA1 can also delay the growth of tumors *in vivo* (Zhang et al., 2020a).

### 3.3 Thyroid cancer

Thyroid cancer is the most common type of endocrine malignant cancer worldwide, and early diagnosis and treatment are critical for improving the prognosis of thyroid cancer patients (Schneider and Chen, 2013; Hao et al., 2021). The identification of ferroptosis-related circRNAs is providing new directions for the early diagnosis and treatment of thyroid cancer (Table 4).

CircKIF4A has been reported to inhibit ferroptosis and promote the malignant progression of papillary thyroid cancer; hence, this circRNA could be targeted in a therapeutic strategy and/or potentially be used as a diagnostic biomarker for thyroid cancer (Chen et al., 2021b). CircKIF4A was found to be highly expressed in papillary thyroid cancer cells, and deletion of circKIF4A inhibited the growth of tumors *in vivo* (Chen et al., 2021b). Similarly, circ_0067934 is known to be elevated in thyroid cancer tissues and inhibits ferroptosis and promotes the progression of thyroid cancer, making it a candidate target and prognosis biomarker for thyroid cancer (Wang et al., 2019c; Wang et al., 2021a). Silencing of circ_0067934 was found to inhibit the growth of tumors *in vivo*, and elevated circ_0067934 was found to be associated with a poor prognosis in thyroid cancer (Wang et al., 2019c). Therefore, targeting circ_0067934 may be a potential therapeutic strategy for regulating ferroptosis in thyroid cancer cells.

### 3.4 Gastric cancer

GC is one of the most harmful cancers in world; it ranks fifth in terms of morbidity rate and fourth in terms of mortality rate (Karimi et al., 2014; Sung et al., 2021). Circ_0000190 induces apoptosis and ferroptosis in GC cells and thus has great potential as a diagnostic and prognostic marker for GC. The expression of circ_0000190 is significantly decreased in GC tissues, and low expression of circ_0000190 was found to be related to the advanced tumor, node, metastasis (TNM) stages of GC (Jiang et al., 2022). In one study, the area under a receiver operating characteristic (ROC) curve of circ_0000190 in GC tissues and plasma was reported to be up to 0.75 and 0.60, respectively (Chen et al., 2017b). Low expression of circ_0000190 is associated with poor survival in GC patients and can be used as a poor prognostic indicator for GC patients (Jiang et al., 2022). Circ_0000190 suppresses GC tumor growth *in vivo*, so restoration of circ_0000190 or ZNRF3 expression may be an effective strategy for GC treatment (Jiang et al., 2022).

### 3.5 Lung cancer

Lung cancer is the second most commonly diagnosed cancer and the leading cause of cancer deaths (Zappa and Mousa, 2016). NSCLC comprises 85% of all lung cancer cases and includes three types of cancer: squamous cell carcinoma, LUAD, and large-cell carcinoma (Zappa and Mousa, 2016).

As inhibitors of ferroptosis, circDTL and circP4HB may prove to be useful diagnostic biomarkers and therapeutic targets for NSCLC. The expression levels of circDTL and circP4HB are significantly increased in NSCLC tissues (Shanshan et al., 2021; Pan et al., 2022). Silencing of circDTL has been shown to improve the sensitivity of NSCLC to chemotherapeutic drugs and inhibit the growth of tumors *in vivo* (Shanshan et al., 2021), and overexpression of circP4HB has been shown to promote tumor growth *in vivo* (Pan et al., 2022). In addition, circP4HB expression is related to the prognosis of patients: the higher the expression of circP4HB, the lower the overall survival rate of patients (Pan et al., 2022).

CircRNA_101093 also has great potential as a diagnostic marker for LUAD. The expression of circRNA_101093 in LUAD tissues and in the plasma exosome of LUAD patients is significantly increased compared to that of healthy individuals, and reducing the exosome improved the outcome of a ferroptosis-based treatment in preclinical *in vivo* models (Zhang et al., 2022a). Improving the efficacy of ferroptosis by blocking exosomal biosynthesis may prove to be a useful strategy for developing ferroptosis-based therapy, and it may also provide a new direction for the future treatment of LUAD (Wang et al., 2022b).

### 3.6 Hepatocellular carcinoma

HCC is one of the most common cancers in the world. It can rapidly develop into a malignant form and has a low 5-year survival rate of <5% (Forner et al., 2012; Lu et al., 2016). Fortunately, circIL4R, an inhibitor of ferroptosis, has potential as a therapeutic target and as a diagnostic and prognostic biomarker for HCC. CircIL4R is significantly upregulated in HCC cells, and deletion of circIL4R has been shown to inhibit tumor growth *in vivo* (Xu et al., 2020). Also, circIL4R has clinical significance in the prognosis of HCC patients: compared with patients with lower expression of circIL4R, patients with higher expression of circIL4R tend to have a lower overall survival rate (Xu et al., 2020).

Circ0097009 is another potential diagnostic biomarker and therapeutic target for HCC. It has been shown that circ0097009 is significantly upregulated in HCC cells and that inhibition of circ0097009 suppresses tumor growth and reduces the number of lung metastases (Lyu et al., 2021). In addition, hsa_circ_0008367, a promoter of ferroptosis in HCC cells treated with SF, is another promising target for improving the cellular sensitivity to SF during HCC treatment (Liu et al., 2020).

### 3.7 Cervical cancer

Cervical cancer is the fourth most common type of malignant tumor in females, and the identification of ferroptosis-related circRNAs provides new opportunities for early diagnosis and treatment of cervical cancer (Li et al., 2021b).

CircEPSTI1, a ferroptosis inhibitor, is a potential therapeutic target and an ideal biomarker for monitoring and treating cervical cancer. CircEPSTI1 expression was found to be upregulated in cervical cancer cell lines, and circEPSTI1 knockdown was found to reduce tumor weight and tumor volume and thus affect the proliferation of cervical cancer cells *in vivo* (Wu et al., 2021).

The identification of circLMO1 as a ferroptosis promotor is also providing new opportunities to develop a therapeutic strategy and a diagnostic and prognostic biomarker for cervical cancer. CircLMO1 has been shown to be downregulated in cervical cancer tissues and to have a negative relationship with the international federation of gynecology and obstetrics (FIGO) stages of cervical cancer (Wu et al., 2021). In addition, overexpression of circLMO1 inhibits cervical cancer cell growth and metastasis both *in vitro* and *in vivo* (Wu et al., 2021).

### 3.8 Colorectal cancer

Globally, colorectal cancer is the third most commonly diagnosed malignancy and the second leading cause of death. Colorectal cancer is a heterogeneous disease that exhibits distinct molecular characteristics in different patient populations (Pawlik, 2022).

Circ_0007142, as a ferroptosis inhibitor, is a promising therapeutic target and potential diagnostic biomarker for colorectal cancer. In colorectal cancer tissues, circ_0007142 has been found to be significantly upregulated, and silencing circ_0007142 has been shown to repress tumorigenesis *in vivo* (Wang et al., 2021b). In addition, higher circ_0007142 expression is associated with larger tumor size, higher TNM classification, distant metastasis, and lymph node metastasis in colorectal cancer patients (Wang et al., 2021b).

CircABCB10 also has great potential as a diagnostic biomarker and therapeutic target for rectal cancer. In a study that involved rectal cancer tissue, circABCB10 was found to be upregulated (Xian et al., 2020). Furthermore, knockdown of circABCB10 promoted ferroptosis and apoptosis in rectal cancer cells *in vitro* and inhibited tumor growth *in vivo* (Xian et al., 2020).

### 3.9 Oral squamous cell carcinoma

OSCC is a very aggressive form of cancer (most patients die within three to 5 years of diagnosis) that affects more than 275,000 people worldwide each year (Pena-Oyarzun et al., 2020). CircFNDC3B is an inhibitor of ferroptosis and promotes the malignant progression of OSCC by regulating the miR-520d-5p/SLC7A11 axis; hence, studies of this circRNA have revealed several potential therapeutic targets and diagnostic and prognostic markers for OSCC (Yang et al., 2021). The expression of both circFNDC3B and *SLC7A11* is enhanced in clinical OSCC tissues, whereas the expression of miR-520d-5p is reduced, and the silencing of circFNDC3B inhibits tumor growth *in vivo* (Yang et al., 2021). In addition, the expression of circFNDC3B in clinical OSCC tissues was found to be negatively correlated with the prognosis of OSCC patients (Yang et al., 2021).

### 3.10 Esophageal cancer

Esophageal cancer is the seventh most frequently diagnosed cancer, and due to its poor prognosis, it is the sixth leading cause of cancer-related death worldwide (Yu et al., 2018; Ajani et al., 2019). Therefore, the discovery of susceptibility genes or new biomarkers is of great significance for the treatment of patients.

CircPVT1 regulates the chemosensitivity of ESCC cells by influencing ferroptosis and the Wnt/b-catenin pathway via the miR-30a-5p/FZD3 axis (Yao et al., 2021). It has been found that circPVT1 expression is enhanced in clinical ESCC tissues (Zhong et al., 2019) and that knockdown of circPVT1 enhances the chemosensitivity of 5-FU-resistant ESCC cells *in vivo* and *in vitro* (Frazer and Anderson, 2014). Thus, circPVT1 is a potential biomarker for ESCC diagnosis and treatment.

### 3.11 Acute lymphoblastic leukemia

ALL occurs in both children and adults, and the prognosis is poor in elderly patients and those with relapsed or refractory ALL (Malard and Mohty, 2020). Therefore, there is a need to develop and implement new diagnostic and therapeutic strategies for this condition. As an inhibitor of ferroptosis that acts via the miR-494-3p/NET1 axis, circ_0000745 is a potential biomarker for the diagnosis and treatment of ALL (Yang et al., 2022). Circ_0000745 expression was found to be significantly upregulated in the peripheral blood samples of patients with acute lymphoblastic leukemia (Yang et al., 2022).

### 3.12 Myocardial infarction

MI is the main cause of sudden cardiac death (Feng and Feng, 2021). It has been found that ferroptosis inhibitors can reverse the effect of ferroptosis in an MI mouse model and improve the survival rate of myocardial cells (Li et al., 2021a). Hence, ferroptosis is a new potential target in the prevention and treatment of MI. CircRNA1615 prevents LRP6-mediated autophagy-related ferroptosis in cardiomyocytes via adsorption of miR-152-3p and controls the pathological process of MI (Li et al., 2021a), providing a potential target for the treatment of MI.

### 3.13 Heart failure

HF is a complex syndrome with a high mortality rate (Zhang et al., 2017). The prognosis of patients with HF is generally poor (Zhang et al., 2017). Therefore, it is necessary to identify and develop appropriate treatment strategies to improve the prognosis and quality of life of HF patients (Zhang et al., 2017). Using an HF mouse model, it has been shown that decreased expression of GPX4 and increased expression of NADPH oxidase 1 and ACSL4 are indicative of lipid peroxidation in cardiomyocytes (Zheng et al., 2021b). Hence, studying circSnx12, a ferroptosis-related circRNA present in cardiomyocytes, may provide new insights into HF and new directions for the development of diagnostic markers or treatments.

### 3.14 Acute cerebral infarction

ACI, also known as ischemic stroke, is the second leading cause of death globally (He et al., 2022). Timely diagnosis and treatment after disease onset, as well as evaluation of the treatment, is the key to saving patients who have experienced an ACI. Despite the progress that has been made in ACI diagnosis and treatment, there is still a need for new methods to increase diagnostic and therapeutic accuracy and efficiency.

Circ-Carm1, which is highly expressed in the serum of ACI patients, promotes the development of ACI via ferroptosis (Xiao et al., 2021). Thus, inhibition of ferroptosis and induction of a circ-Carm1 deficiency may be a promising approach for the prevention and treatment of ACI.

### 3.15 Traumatic brain injury

Globally, TBI is the leading cause of death, and more than 60 million people experience TBI each year (Dewan et al., 2019). Moreover, TBI has been associated with a long-term risk of neurological disease (Turner et al., 2021). CircPtpn14 is a ferroptosis promoter and opposes the therapeutic effect that melatonin has in TBI cases via the miR-351-5p/5-LOX signaling pathway. Hence, circPtpn14 is a potential target in TBI treatment strategies.

### 3.16 Polycystic ovary syndrome

PCOS is one of the most common endocrine and metabolic disorders in premenopausal women. It is characterized by a series of signs and symptoms, namely, clinical or biochemical hyperandrogenism, oligoovulation, and polycystic ovarian morphology (Azziz, 2018; Escobar-Morreale, 2018). CircRHBG inhibits ferroptosis in PCOS cells and thus should be investigated as a potential diagnostic molecular marker and therapeutic target for PCOS (Zhang et al., 2021a). In the granulosa cells of PCOS patients, circRHBG expression was found to be significantly upregulated, and circRHBG knockdown can inhibit cell proliferation and decrease cell viability (Zhang et al., 2021a).

### 3.17 Diabetic nephropathy

About 40% of people with diabetes develop DN (Gross et al., 2005). Extensive innovations are urgently needed to improve the health outcomes of patients with DN. In terms of the use of circRNAs, the efficacy of exogenous mmu_circRNA_0000309 in combination with germacrone should be examined as a potential DN treatment. Given that germacrone inhibits ferroptosis-dependent mitochondrial damage and podocyte apoptosis by regulating the miR-188-3p/GPX4 axis in combination with exogenous mmu_circRNA_0000309, such studies would provide insight into the potential of this combination as a treatment for DN (Jin et al., 2022).

### 3.18 Diabetic retinopathy

More than 45% of people with type 2 diabetes have DR, which is the leading cause of blindness in adults (Calderon et al., 2017). In most cases, DR is not noticed until it irreversibly damages the eye and causes blurred vision and eventual blindness (Adki and Kulkarni, 2020). Therefore, early diagnosis is vital for the treatment of patients with DR. Circ-PSEN1 regulates ferroptosis in retinal pigment epithelial cells of patients with DR via the miR-200b-3p/CFL2 axis and thus may be a novel therapeutic target for DR.

### 3.19 The incorporation of circRNA data into machine learning models to identify therapeutic targets and diagnostic and prognostic biomarkers

Machine learning is an indispensable tool for identifying relevant biomarkers and classifying samples in the validation of biomarkers (Zhang et al., 2020b; Chen et al., 2022b). CircRNAs, as potential biomarkers of various diseases, have been widely incorporated into machine learning models for disease diagnosis, treatment, and prognosis prediction. As a result, machine learning classification models have identified several circRNAs as potential disease biomarkers, such as circERBB2 and circCHST12 for intracerebral hemorrhage diagnosis (Bai et al., 2022), circ-0080695 for liver cancer diagnosis (Zhu et al., 2021b), circ_0059706 for acute myeloid leukemia prognosis (Ma et al., 2022b), and hsa_circ_0007919, hsa_circ_0002419, and hsa_circ_0005521 for pulmonary tuberculosis diagnosis (Yuan et al., 2022).

In addition to conventional logistic regression, gradient boosting, deep neural networks, and K-means clustering algorithms, some useful new models and frameworks have also been used to predict circRNA–disease associations, such as SGANRDA (Wang et al., 2021c), MRLDC (Xiao et al., 2019) and MSFCNN (Fan et al., 2020), GCNCDA (Wang et al., 2020b), MDGF-MCEC (Wu et al., 2022b), CLCDA (Wang et al., 2023), and GBDTCDA (Lei and Fang, 2019).

In terms of the statistical tools used, ROC curve analysis has typically been used to examine the potential diagnostic value and investigate the specificity and sensitivity of the identified circRNAs as diagnostic biomarkers. Kaplan–Meier survival curve analysis has generally been used to examine the potential prognostic value of the identified circRNAs.

Using machine learning tools to further predict the associations among the abovementioned ferroptosis-related circRNAs, diseases, and ferroptosis may provide researchers in the field with an effective and efficient method for generating reliable classification criteria for the clinical application of these potential disease biomarkers.

## 4 Perspective

Ferroptosis is a lipid peroxidation-driven and iron-dependent form of cell death (Chen et al., 2021a). This unique form of cell death is regulated by a variety of cellular metabolic pathways, such as redox homeostasis, iron treatment, mitochondrial activity, and metabolism of amino acids, lipids, and sugars (Jiang et al., 2021). Many organ injuries and degenerative lesions are driven by ferroptosis (Jiang et al., 2021).

CircRNA is a newly identified class of non-coding single-stranded RNA without free 3′poly (A) tails or 5′caps (Ren et al., 2020). CircRNA is abundant in eukaryotes, conserved in evolution, highly stable, and tissue-specific; it also plays crucial roles in many tissue types (Xu et al., 2017; Kristensen et al., 2019; Chen, 2020). Due to their characteristics, circRNAs have great potential as biomarkers in tumor diagnosis and as targets in tumor treatment.

In this review, we have outlined the recent progress made in understanding the roles of circRNAs in the molecular mechanisms and regulatory networks of ferroptosis and the potential clinical applications of circRNAs in ferroptosis-related diseases. More than 20 circRNAs have been reported to inhibit ferroptosis by acting on GPX4, system Xc-, FSP1, lipid metabolism, and other pathways and play important regulatory roles in the progression of many diseases, including various cancers, diabetic nephropathy, polycystic ovary syndrome, and myocardial infarction. Seven circRNAs have been reported to stimulate ferroptosis and play important regulatory roles in the progression of cervical cancer, acute cerebral infarction, traumatic brain injury, diabetic retinopathy, hepatocellular carcinoma, and gastric cancer. These ferroptosis-related circRNAs have great potential as biomarkers in the diagnosis, treatment, and prognosis of diseases. This review furthers our understanding of the roles of ferroptosis-related circRNAs and provides new perspectives on ferroptosis regulation and new directions for the diagnosis, treatment, and prognosis of ferroptosis-related diseases.

Notably, the research on circRNAs in ferroptosis is still incomplete. Most of the recently published studies on ferroptosis-related circRNAs were conducted with tumor tissues and cells; therefore, using blood, urine, or tear samples in future studies may provide new insights and ideas for further research. It is also likely that there are many more ferroptosis-related circRNAs that have not yet been discovered. The circRNAs that are found to be biomarkers of ferroptosis may provide new perspectives for the detection of ferroptosis. However, the notion that ferroptosis-related circRNAs can be used as biomarkers of ferroptosis must also be further interrogated.

The ultimate goal of conducting all the studies described in this review is to improve clinical disease diagnosis and treatment. However, most of the studies have been conducted under experimental conditions. Thus, there is a need to undertake a large number of clinical studies and experiments to ensure the safety and efficacy of the tested molecules and methods.

Although there are still many obstacles hindering our efforts to explore the potential of ferroptosis-related circRNAs in the diagnosis and treatment of diseases, we believe that understanding the interactions between circRNAs and ferroptosis will help us to address these barriers. Based on the progress made to date, it is clear that circRNAs related to ferroptosis will be widely used in the diagnosis, treatment, and prognosis of diseases and in research on drug resistance in the future. These advances will greatly reduce mortality rates and improve cure rates, alleviating the pain of patients and bringing happiness to their lives.
